# The impact of genetic relationship information on genomic breeding values in German Holstein cattle

**DOI:** 10.1186/1297-9686-42-5

**Published:** 2010-02-19

**Authors:** David Habier, Jens Tetens, Franz-Reinhold Seefried, Peter Lichtner, Georg Thaller

**Affiliations:** 1 Institute of Animal Breeding and Husbandry, Christian-Albrechts University of Kiel, Olshausenstrasse 40, 24098 Kiel, Germany; 2 Vereinigte Informationssysteme Tierhaltung w.V., Heideweg 1, 27283 Verden/Aller, Germany; 3 Helmholtz Zentrum München, German Research Center for Environmental Health, Ingolstädter Landstrasse 1, 85764 Neuherberg, Germany

## Abstract

**Background:**

The impact of additive-genetic relationships captured by single nucleotide polymorphisms (SNPs) on the accuracy of genomic breeding values (GEBVs) has been demonstrated, but recent studies on data obtained from Holstein populations have ignored this fact. However, this impact and the accuracy of GEBVs due to linkage disequilibrium (LD), which is fairly persistent over generations, must be known to implement future breeding programs.

**Materials and methods:**

The data set used to investigate these questions consisted of 3,863 German Holstein bulls genotyped for 54,001 SNPs, their pedigree and daughter yield deviations for milk yield, fat yield, protein yield and somatic cell score. A cross-validation methodology was applied, where the maximum additive-genetic relationship (*a*_*max*_) between bulls in training and validation was controlled. GEBVs were estimated by a Bayesian model averaging approach (BayesB) and an animal model using the genomic relationship matrix (G-BLUP). The accuracy of GEBVs due to LD was estimated by a regression approach using accuracy of GEBVs and accuracy of pedigree-based BLUP-EBVs.

**Results:**

Accuracy of GEBVs obtained by both BayesB and G-BLUP decreased with decreasing *a*_*max *_for all traits analyzed. The decay of accuracy tended to be larger for G-BLUP and with smaller training size. Differences between BayesB and G-BLUP became evident for the accuracy due to LD, where BayesB clearly outperformed G-BLUP with increasing training size.

**Conclusions:**

GEBV accuracy of current selection candidates varies due to different additive-genetic relationships relative to the training data. Accuracy of future candidates can be lower than reported in previous studies because information from close relatives will not be available when selection on GEBVs is applied. A Bayesian model averaging approach exploits LD information considerably better than G-BLUP and thus is the most promising method. Cross-validations should account for family structure in the data to allow for long-lasting genomic based breeding plans in animal and plant breeding.

## Background

The development of high-throughput genotyping of single nucleotide polymorphisms (SNPs) has enhanced the use of genome-wide dense marker data for genetic improvement in livestock. Meuwissen et al. [1] presented a two-step approach to predict genomic breeding values (GEBVs): First, SNP effects are estimated using genotyped individuals that are phenotyped for the quantitative trait (training), and then GEBVs are predicted for any genotyped individual by using only its SNP genotypes and estimated SNP effects. This prediction and selection on GEBVs was termed genomic selection (GS).

The acceptance of GS by cattle breeders and thereby the potential to reduce generation intervals depends mainly on the accuracy of GEBVs. Assuming that cosegregation is not modeled, GEBV accuracy is higher than the accuracy of standard pedigree-based BLUP-EBVs only if there is linkage disequilibrium (LD) between SNPs and quantitative trait loci (QTL). LD is defined here as the dependency between the allele states at different loci of all individuals in the available data set. In case of linkage equilibrium, the accuracy of GEBVs is not necessarily zero but will approach the accuracy of pedigree-based BLUP-EBVs as the number of SNPs fitted in the model increases. The reason is that SNPs capture additive-genetic relationships irrespective of the amount of LD in the population as demonstrated by Habier et al. [2] and Gianola et al. [3]. In those studies as well as here, additive-genetic relationships are defined as twice the coefficient of coancestry given by Malécot [4]. Note that this does not require that the training individuals are related, but only that individuals for which GEBVs are estimated are related to the training individuals. This is demonstrated in detail in additional file 1 in this paper. In practice, LD exists in cattle populations [5-7] and thus two types of information are utilized to estimate GEBVs: LD and additive-genetic relationships. If cosegregation is modeled, then a third type of information can be utilized. However, cosegregation was not modeled in this study. The persistence of the accuracy of GEBVs over generations, and therefore the potential of GS to reduce future phenotyping [8,9], depends largely on the amount of LD, which originates in outbred populations from historic mutations and drift, cosegregation, migration, selection and recent drift. In simulations, Habier et al. [2] estimated the accuracy of GEBVs that is only due to LD (in short, accuracy due to LD), which was considerably smaller than the GEBV accuracy resulting from both LD and additive-genetic relationships in the offspring of the training individuals, but it was fairly persistent over generations. Furthermore, the ability to exploit LD information by the statistical methods used to estimate SNP effects varies. Meuwissen et al. [1] proposed a Bayesian model averaging approach termed BayesB, which fits only a small proportion of the available SNPs in each round of a Markov-Chain Monte Carlo (MCMC) algorithm and models SNP effects with a t-distributed prior. They further used Ridge-Regression BLUP (RR-BLUP), which fits all SNP effects with a normal prior. Habier et al. [2] showed that BayesB was more able to exploit LD information and less affected by additive-genetic relationships than RR-BLUP. Accuracy of GEBVs from real cattle data has been reported for Holstein Friesian populations from North America [10], Australia, the Netherlands and New Zealand [11,12]. In those studies, accuracies of GEBVs for milk performance, fertility and functional traits ranged from 0.63 to 0.84, and depended on the size of the training data, heritability and SNP density. These accuracies confirmed those found in simulations [1,2,13,14] quite well, but RR-BLUP was only slightly inferior compared to methods that fit only a fraction of the available SNPs such as BayesB. VanRaden et al. [10] and Hayes et al. [12] concluded that, unlike in most simulations, only a few QTL with a large effect and many with a small effect contribute to genetic variation. These studies, however, did not show the dependency of the GEBV accuracy on additive-genetic relationships, which is a function of the number of relatives in training, the degree of relationship with training individuals [2] and heritability. Thus, a lower accuracy with decreasing training size [10] could be the result of a lower number of relatives in training, meaning that the more persistent accuracy due to LD and the GS method that exploits LD information best remains to be evaluated for real cattle data. More important, the dependency of GEBV accuracy on additive-genetic relationships as well as the accuracy due to LD must be known to develop future breeding programs, because close relatives that were progeny tested for quantitative traits may not be available when GEBVs are applied to select animals early in lifetime. The objectives of this study were to analyze the impact of additive-genetic relationships between training and validation data sets on the accuracy of GEBVs and to estimate the accuracy due to LD in the German Holstein Friesian population. Thereby, the accuracy of GEBVs for current and future selection candidates as well as for individuals that are unrelated to the population were estimated. Furthermore, the comparison of BayesB and RR-BLUP based on the accuracy due to LD will show which statistical model has the potential to reduce future phenotyping.

## Materials and methods

### Genotyped bulls

A total of 3,863 German Holstein Friesian bulls, progeny tested with at least 30 daughters in the first lactation and genotyped for 54,001 SNPs distributed over the whole genome, were available. The proportion of missing genotypes for any bull was lower than 5%, at an average of 1%. The distribution of genotyped bulls by birth year and the average number of daughters per bull are shown in Table 1. The family structure of these bulls, consisting of paternal half and full sib families as well as genotyped fathers and sons, are summarized in Table 2.

**Table 1:** Distribution of genotyped bulls (*n *= 3,863) by birth year and average number of phenotyped daughters per bull (s.e.)

Birth year	No. of bulls	No. of daughters
1981-1989	140	5,969 (± 886)
1990-1997	455	4,473 (± 356)
1998	378	567 (± 115)
1999	446	297 (± 42)
2000	484	142 (± 11)
2001	482	107 (± 2)
2002	485	116 (± 2)
2003	830	93 (± 1)
2004	163	62 (± 2)

**Table 2:** Family structure of genotyped bulls. Number (*n*), average size (), standard deviation (*s*), minimum (Min) and maximum (Max) size of paternal half and full sib families as well as number of genotyped fathers and summary statistics for the number of their genotyped sons

Family type	*n*		*s*	Min	Max
Half sib	646	6.0	10.9	1	102
Full sib	168	2.2	0.4	2	5
Father-son	114	10.7	19	1	109

### SNP data

DNA was extracted either from frozen semen, leukocyte pellets or fullblood samples. The BovineSNP50 BeadChip (Illumina, San Diego, CA) was used to obtain SNP genotypes for all bulls. A detailed description of the SNP content was given by [15]. Only SNPs with less than 5% missing genotypes and minor allele frequency greater than 3% were used, resulting in 40,588 SNPs. Minor allele frequencies of the selected SNPs were nearly uniformly distributed with a mean = 0.27. Genotypes of SNPs located on the X chromosome, but outside the pseudo-autosomal region, were set to missing if the genotype of a bull was heterozygous. Missing genotypes were imputed by *fastPhase *[16].

Furthermore, the haplotypes obtained by *fastPhase *were utilized in Haploview [17] to estimate *r*^2 ^as a measure of LD between SNPs. Haplotypes of all genotyped bulls were used in this calculation, because the aim was to evaluate the LD that can be utilized to estimated SNP effects, and this LD may have also been caused by cosegregation, recent drift and selection.

### Pedigree information

The pedigree consisted of genotyped bulls as well as their ancestors born between 1950 and 1998, yielding a total of 21,591 individuals. This pedigree was used to generate training and validation data sets with a specified maximum additive-genetic relationship between bulls in both data sets and to estimate breeding values with the standard BLUP-methodology.

### Phenotypes

Daughter yield deviations (DYDs) [18] for the quantitative traits milk yield, fat yield, protein yield and somatic cell score were available for both genotyped bulls and their male ancestors in the pedigree. They were estimated from the test-day yields of daughters corrected for fixed and permanent environmental effects as well as half the breeding value of the daughter's dam [19]. Phenotypes and estimated effects were taken from the April 2009 evaluation for the German Holstein Friesian population. Additive-genetic and residual variances,  and , used as prior information in the statistical analyses, were estimated by ASReml [20] utilizing all phenotyped bulls in the pedigree. A sire model was used for this purpose in which residual terms were weighted by the reliability of a bull's DYD.

### Statistical models

Three statistical models were used to evaluate the impact of additive-genetic relationships on the accuracy of GEBVs. These were 1) BayesB [1], 2) BLUP animal model using the genomic relationship matrix [21], which is equivalent to RR-BLUP [2,22,23], and 3) BLUP animal model using the numerator relationship matrix [24,25] to estimate standard BLUP breeding values. These models are described in more detail below.

The statistical model for BayesB can be written as

where *y*_*i *_is the DYD of bull *i *in training, *α *is an intercept, *K *= 40, 588 SNPs, *x*_*ik *_is the SNP genotype, *β*_*k *_is the effect and *δ*_*k *_is a 0/1-indicator variable, all for SNP *k*, *e*_*i *_is the residual effect with mean zero and variance , and *w*_*i *_is the reliability of *y*_*i*_. SNP genotypes are coded as the number of copies of one of the SNP alleles, i.e. 0, 1 or 2. The prior for *α *was 1, for  scaled inverse chi-square with degrees of freedom *ν*_*e *_= 4.2 and scale , and for *δ*_*k *_the probability that SNP *k *is fitted in the model, *π *= Pr(*δ*_*k *_= 1), which was set to 0.01. SNP effects are treated as random and are sampled from *N *(0, ), where  has a scaled inverse chi-square prior with *ν*_*β *_= 4.2 and . The variance  was calculated as , where *p*_*k *_is the allele frequency at SNP *k*. MCMC-sampling was used to infer model parameters, where *α*, *β*_*k *_and  were sampled with Gibbs steps and *δ*_*k *_and  with a Metropolis-Hastings step. The MCMC-sampler was run for 50,000 iterations with a *burn-in *of 40,000 rounds. The GEBV of bull *i*, either in training or validation, was estimated as(1)

where  is the estimated SNP effect of locus *k*. The BLUP animal model used to estimate genomic or pedigree-based EBVs is

where *y*_*i*_, *e*_*i *_and *w*_*i *_are defined as before, *μ *is the overall mean, and *g*_*i *_is the breeding value of bull *i *in training. Genomic BLUP (G-BLUP) EBVs of both training and validation bulls were obtained by mixed-model equations using the genomic relationship matrix, whereas pedigree-based BLUP (P-BLUP) EBVs were obtained by using the numerator relationship matrix [24,25]. The elements of the genomic relationship matrix were calculated as  following [2,26], where ***x***_*k *_is a column vector containing the SNP genotypes of training and validation bulls at locus *k*.

### Generation of training and validation data sets

Training and validation data sets were generated systematically using the additive-genetic relationships between bulls derived from the pedigree in order to study the impact of additive-genetic relationship information on accuracy of GEBVs by cross-validation. The aim was to control the maximum additive-genetic relationship between bulls in training and validation denoted by *a*_*max*_; That is, given *a*_*max*_, no bull in training was allowed to have an additive-genetic relationship larger than *a*_*max *_with a bull in validation. This criterion allows to divide the family structure present in the data set such that validation bulls are allowed to have close relatives in training or not. Furthermore, the decay of additive-genetic relationships over generations, similar to that in simulation studies [1,2,14,27], can be mimicked. A sampling algorithm was implemented to generate training and validation data sets, which assigned bulls to both sets in a way that *a*_*max *_was not exceeded. For small *a*_*max *_values this can only be achieved by removing completely some bulls from the analysis, where the algorithm was optimized to exclude as few bulls as possible.

In general, the lower the *a*_*max*_, the smaller the number of bulls in validation. Therefore, several pairs of training and validation data sets were sampled, where repeated sampling of a bull into validation was not accepted. In addition, no more than two bulls out of one half sib family were allowed to be in validation in order to reduce the dependency between validation bulls in each pair of data sets. Furthermore, fathers of training bulls were not allowed to be in validation, because the accuracy of those bulls is not representative for the prediction of the GEBVs of future individuals as demonstrated by [2].

### Relationships between training and validation data sets

Four different scenarios with *a*_*max *_= 0.6, 0.49, 0.249 and 0.1249 were generated. These values were selected to exploit the family structure in the data as follows: The training data set contained fathers, full- and half sibs of the bulls in validation with *a*_*max *_= 0.6, only half sibs with *a*_*max *_= 0.49, and neither of those close relatives with *a*_*max *_= 0.249 and 0.1249. All scenarios had the same training size in order to exclude the effect of different sizes on the accuracy of GEBVs. Because of difficulties to obtain large training data sets for the lowest *a*_*max *_in a structured dairy cattle population, the training sizes for the other scenarios were reduced to the average size of *a*_*max *_= 0.1249 by removing bulls randomly. Note, however, that for the scenarios with *a*_*max *_= 0.6 and 0.49 the half and full sibs or fathers of the bulls in validation were not removed from the training data. The training size for *a*_*max *_= 0.1249 was 2,096 bulls on average in 15 sampled pairs of training and validation data sets, hence the training size for the other scenarios was fixed at 2,096 bulls. Validation data sets of each sample for the first three scenarios were required to have at least 30 bulls, and for *a*_*max *_= 0.1249 at least 11 bulls. The correlation between EBVs and DYDs was also estimated for training bulls and denoted as scenario *a*_*max *_= 1.

To study the effect of the size of the training data on accuracy at different *a*_*max *_values, training data sets were halved to a size of 1,048 by removing bulls randomly, except for fathers as well as full and half sibs of the bulls in validation. Thus, the number of close relatives between training and validation was kept constant in order to analyze the impact of the precision of SNP effects on accuracy rather than the number of relatives, which can already be observed with decreasing *a*_*max*_.

### Criterion for comparisons

The correlation between true and estimated breeding values, *g *and , was estimated by the following formula:

where *y *denotes DYD,  the heritability of DYDs and  the correlation between the true breeding value and DYD averaged over bulls in validation. The latter was estimated from the accuracy of DYDs using the selection index formula , where *n*_*i *_is the number of daughters of a bull *i *and  the heritability of a daughter record of trait *j *known from parameter estimations by Liu et al. [28,29]. The heritabilities for milk, fat and protein yield as well as somatic cell score were 0.53, 0.52, 0.51 and 0.23, respectively. The correlation  was calculated using the validation bulls from all replicates of a specified *a*_*max*_, after their EBVs were corrected by the mean EBV of their respective validation data set.

### Accuracy due to LD

The accuracy due to LD was estimated using a regression approach as suggested by Habier et al. [2]. In that study, the authors estimated the accuracy due to LD of generation *j*, , by using the accuracy of GEBVs obtained from four generations and the model

where *ρ*_*i *_is the accuracy of GEBVs in generation *i*, *x*_1*i *_is the accuracy of P-BLUP in generation *i *divided by the accuracy of P-BLUP in generation *j*, which models the decay of P-BLUP accuracy due to the decline of additive-genetic relationships, *d*_*j *_is the difference between the accuracy of GEBVs and the accuracy due to LD in generation *j*, *x*_2*i *_is the decay of LD over generations and *e*_*i *_is a residual term. In this study, accuracies of GEBVs from different generations were replaced by those from different *a*_*max *_values. Furthermore, the accuracy due to LD was assumed to be constant with different *a*_*max *_values, because the average birth year of training and validation bulls was nearly the same for all *a*_*max *_values and thus *x*_2*i *_is always 1. The equation used here was(2)

where  is the accuracy of GEBVs for *a*_*max *_(i.e., 0.6, 0.49, 0.249 and 0.1249) estimated by BayesB or G-BLUP,  is the accuracy of P-BLUP for *a*_*max *_divided by the accuracy of P-BLUP for *a*_*max *_= 0.6, *d *is the difference between the accuracy of GEBVs for *a*_*max *_= 0.6 and the accuracy due to LD and  is a residual term.

## Results

### Linkage disequilibrium

**Figure 1:**
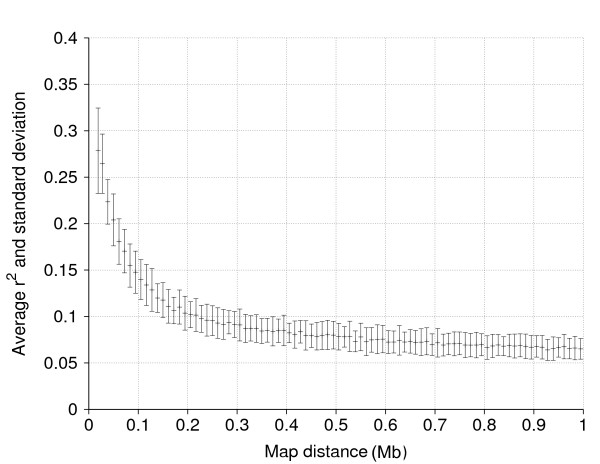
**Average *r*^2 ^(mid-point) as a measure of linkage disequilibrium between syntenic SNP pairs against map distance in megabase (Mb) as well as standard deviation of mean *r*^2 ^values from all 30 chromosomes (upper and lower deviation from the mid-point)**.

Figure 1 shows average *r*^2 ^between syntenic SNP pairs against map distance of up to 1 megabase (Mb), which is roughly 1 centimorgan, as well as standard deviations of the average *r*^2 ^values across all 30 chromosomes. Average *r*^2 ^decreased exponentially with increasing distance between SNPs and was equal to 0.29, 0.23, 0.15 and 0.07 at distances of 0.02, 0.04, 0.1, and 1 Mb, respectively. Average distance and *r*^2 ^of adjacent SNPs were 0.064 Mb and 0.22, respectively.

### Training and validation data

**Figure 2:**
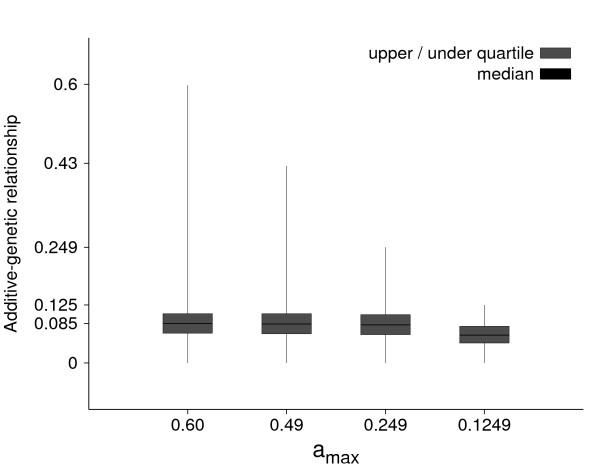
**Box plots of additive-genetic relationships between bulls in training and validation for a maximum additive-genetic relationship, *a*_*max*_, of 0.6, 0.49, 0.249 and 0.1249**.

Table 3 summarizes the number of bulls used for training in each sampled pair of training and validation data sets as well as the total number of validation bulls over all samples for the specified *a*_*max *_values. Fifteen pairs of training and validation data sets were generated for each scenario with an average validation size of 33 bulls per sampled pair for *a*_*max *_= 0.6, 0.49 and 0.249, and 11 bulls for *a*_*max *_= 0.1249. To better understand the differences in the accuracy of GEBVs between *a*_*max *_values in the following description of the results, the distributions of additive-genetic relationships between bulls in training and validation depending on *a*_*max *_are depicted in Figure 2. The scenarios *a*_*max *_= 0.6, 0.49 and 0.249 only differed in the upper parts of their distributions, whereas mean (not shown), median and quartiles were nearly identical. The training data for *a*_*max *_= 0.49 contained half sibs which were not in the training data sets for *a*_*max *_= 0.249 and 0.1249, and the scenario with *a*_*max *_= 0.6 had also full sibs and fathers of bulls in validation which were not in the scenario 0.49. A validation bull had on average 10 half sibs in training in both scenarios with *a*_*max *_= 0.6 and 0.49, but numbers varied largely between 1 and 58. Only a few validation bulls had a full sib or father in training in the scenario with *a*_*max *_= 0.6.

**Table 3:** Average number of bulls used for training in each of the 15 sampled pairs of training and validation data sets and total number of validation bulls over all pairs for a maximum additive-genetic relationship between bulls of both data sets (*a*_*max*_) of 0.6, 0.49, 0.249 and 0.1249

	No. of bulls in	
	
*a*_*max*_	training	validation
0.60	2,096	491
0.49	2,096	497
0.249	2,096	477
0.1249	2,096 (± 28)	176

### Accuracy of GEBVs

**Figure 3:**
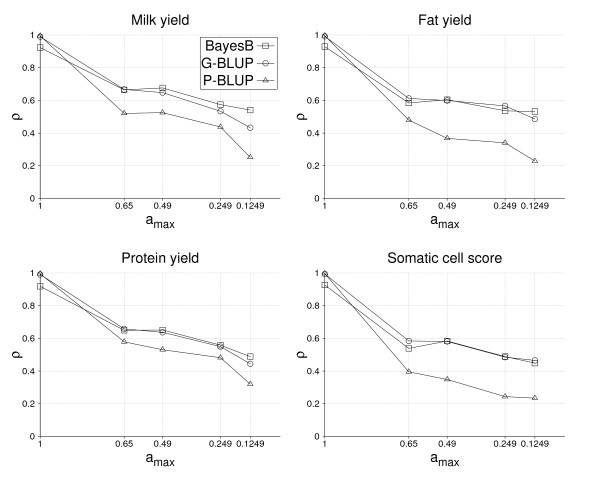
**Accuracy of EBVs, *ρ*, obtained by BayesB, G-BLUP and P-BLUP depending on the maximum additive-genetic relationship between bulls in training and validation, *a*_*max*_, for the traits milk yield, fat yield, protein yield and somatic cell score, based on 2,096 training bulls in each *a*_*max *_scenario**.

Figure 3 depicts the accuracy of EBVs depending on *a*_*max *_for milk, fat and protein yield as well as somatic cell score obtained by BayesB, G-BLUP and P-BLUP utilizing 2,096 training bulls. For *a*_*max *_= 1, accuracies were close to unity for G-BLUP and P-BLUP, but somewhat lower for BayesB. The reason is that accuracies for *a*_*max *_= 1 describe goodness of fit rather than prediction ability and it is well known that the coefficient of determination, which is related to this accuracy, increases with the number of explanatory variables. G-BLUP used all available SNPs, whereas BayesB fitted only 400 in each round of the MCMC-algorithm. Accuracy of P-BLUP decreased with *a*_*max *_as expected, where the overall level for milk and protein yield was higher than for fat yield and somatic cell score. P-BLUP was outperformed by both GS methods, where the absolute difference between the latter and P-BLUP was higher for fat yield and somatic cell score compared to milk and protein yield. The highest accuracies of GEBVs were found for *a*_*max *_= 0.6 and 0.49 and equal to 0.68, 0.65 and 0.60 for milk, fat and protein yield, respectively, and 0.58 for somatic cell score. BayesB and G-BLUP gave similar results in all traits except for milk yield for which BayesB performed notably better. Interestingly, the accuracy of GEBVs from both GS methods was very similar for *a*_*max *_values = 0.6 and 0.49, although a decay was found from 0.6 to 0.49 for P-BLUP in most traits. No plausible reason could be found for that, especially as the decay of accuracy of the GS methods resembled that of P-BLUP quite well otherwise.

Accuracy of GEBVs clearly decreased from *a*_*max *_= 0.49 to 0.1249 in all four traits and for both BayesB and G-BLUP (Figure 3). The decay of accuracy was similar for both GS methods in somatic cell score, but smaller with BayesB for the yield traits. As a result, the accuracy of the yield traits at *a*_*max *_= 0.1249 was higher with BayesB than with G-BLUP. Furthermore, the smallest decay of accuracy was found for fat yield, followed by somatic cell score.

### Accuracy with half the training data

**Figure 4:**
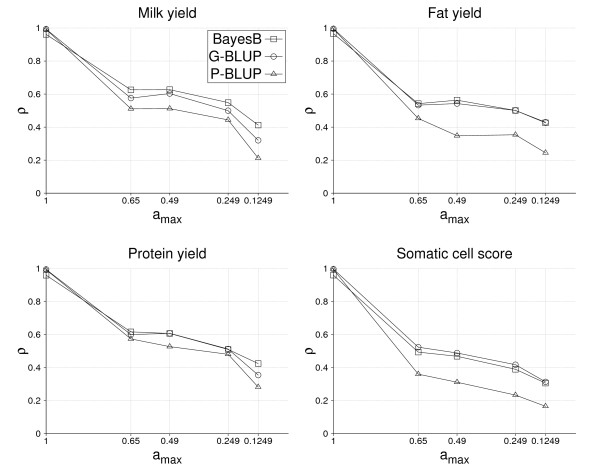
**Accuracy of EBVs, *ρ*, obtained by BayesB, G-BLUP and P-BLUP depending on the maximum additive-genetic relationship between bulls in training and validation, *a*_*max*_, for the traits milk yield, fat yield, protein yield and somatic cell score, based on 1,048 training bulls in each *a*_*max *_scenario**.

With a training size of only 1,048 bulls, the accuracy level of all methods decreased (Figure 3 and 4). Because the number of fathers, half and full sibs of validation bulls was identical for both training sizes analyzed, accuracy of GEBVs for the yield traits decreased by only 0.03 to 0.05 for *a*_*max *_values = 0.6 and 0.49. The loss in accuracy with decreasing *a*_*max *_was similar for both training sizes from *a*_*max *_= 0.49 to 0.249, but considerably larger from 0.249 to 0.1249 with only 1,048 training bulls. The differences between BayesB and G-BLUP were comparable for the two training sizes, except for *a*_*max *_= 0.1249 where differences tend to decrease with the smaller training data set.

### Accuracy due to LD

Table 4 shows the accuracy due to LD estimated by equation (2) for the two sizes of training data sets and the four traits analyzed. With 2,096 training bulls, the accuracy due to LD is always higher for BayesB than for G-BLUP, where the largest difference of 0.2 and 0.12 between methods was obtained for milk and protein yield, respectively, and smallest for somatic cell score. With only half the training size, both the accuracies due to LD and the differences between the two GS methods decreased considerably. The absolute decay of accuracies was similarly high for milk yield, fat yield and somatic cell score, but notably smaller for protein yield, which had the smallest accuracy of all traits with 2,096 training bulls. Furthermore, BayesB and G-BLUP gave very similar accuracies for fat yield and somatic cell score using 1,048 training bulls, whereas BayesB was consistently better for milk and protein yield. In comparison to the accuracies of GEBVs with 2,096 training bulls (Figure 3), differences between BayesB and G-BLUP became more distinct for the accuracies due to LD. In addition, the ranking of the traits according to their accuracies is different. Milk and protein yield had clearly higher accuracies of GEBVs than fat yield and somatic cell score, whereas fat yield had the highest accuracy due to LD and protein yield the lowest.

**Table 4:** Accuracy of GEBVs due to LD estimated by equation (2) for milk, fat and protein yield as well as somatic cell score using training data sizes of 2,096 and 1,048 bulls.

Training data size	Method	Milk yield	Fat yield	Protein yield	Somatic cell score
2,096	BayesB	0.41	0.47	0.29	0.33
	G-BLUP	0.21	0.38	0.17	0.29
1,048	BayesB	0.24	0.31	0.23	0.15
	G-BLUP	0.13	0.33	0.10	0.15

## Discussion

The objective of this study was to analyze the impact of additive-genetic relationships between bulls in training and validation data sets on the accuracy of GEBVs and to estimate the accuracy due to LD. The accuracy of GEBVs obtained by both BayesB and G-BLUP decreased with maximum additive-genetic relationship between bulls in training and validation (*a*_*max*_) for all four traits analyzed. The decay of accuracy tended to be larger for G-BLUP and when training size was smaller. The differences between BayesB and G-BLUP became more evident considering the accuracy due to LD. BayesB clearly outperformed G-BLUP in sets of 2,096 training bulls. The LD found here is comparable to that reported by De Roos et al. [5] for the Dutch and Australian Holstein populations making the results of this study meaningful for other Holstein populations.

### Variability of accuracy of GEBVs

Results of this study demonstrate that the accuracy of GEBVs is not constant for all selection candidates but can vary depending on the number of relatives in training and the degree of additive-genetic relationships with training individuals (Figure 3 and 4). The impact of additive-genetic relationships also depends on the method used to estimate SNP effects [2], because the more SNPs fitted, the more additive-genetic relationships are captured by them. This may explain why G-BLUP tended to decrease more with *a*_*max *_than BayesB. In principle, the decay of accuracy with additive-genetic relationships is also expected to be higher with increasing heritability, but this could not be observed here.

The accuracies of GEBVs reported in this study are representative for the prediction of GEBVs of future generations, in that fathers with offspring in training were not used for validation. Otherwise the accuracy would be higher because their Mendelian sampling terms could be inferred by utilizing the additive-genetic relationships captured by SNPs.

In conclusion, the additive-genetic relationships between training individuals and a selection candidate must be known in order to provide a reliable GEBV accuracy of that candidate in practical application. As was shown with Figure 2, the average additive-genetic relationship for the *a*_*max *_scenarios 0.6, 0.49 and 0.249 did not differ, and thus is not helpful to describe the impact of additive-genetic relationships on accuracy, but rather *a*_*max*_. This criterion was selected here to exploit the family structure in the data, but other criteria should be found that are more useful in practice. One possibility, which should be tested in subsequent studies, could be the expected accuracy of P-BLUP obtained from theoretical calculations.

To evaluate the expected variation in accuracy for young selection candidates, *a*_*max *_was calculated for bulls born in 2007 with respect to the full training data set of 3,863 bulls. Fortunately, all selection candidates have *a*_*max*_≥ 0.125, 83% have *a*_*max*_≥ 0.25, and one third even have ancestors and full sibs in training. The reason for these high genetic relationships are the long generation intervals in cattle and the low effective population size of 40-50 (personal unpublished studies, estimated from pedigree). This shows that the accuracy of GEBVs for current selection candidates is expected to vary due to different additive-genetic relationships with the training data.

### Accuracy due to LD

Accuracy due to LD ranged between 0.29 for protein yield to 0.48 for fat yield using 2,096 training bulls and BayesB. With this number of training bulls, accuracy due to LD, which is expected to be fairly persistent over generations, appears to be too small to reduce trait phenotyping, and progeny testing in particular if GS is applied. However, accuracy due to LD improved considerably with increasing training size and thus further studies are necessary to evaluate the accuracy due to LD with the current training size of 3,863 bulls and beyond. Further improvements may be possible by varying the strong prior probability of fitting a SNP locus into the model, *π*, or by treating it as another variable model parameter.

The accuracy due to LD may be a lower bound for the accuracy of an individual that is unrelated to the training population. However, if LD is primarily due to selection and recent drift rather than historic mutations, the accuracy for unrelated individuals might be even lower. This could be the case if selection candidates descend from a population having an LD structure that is different from that in the training data. This may apply to individuals either from families that did not contribute to the actual German Holstein Friesian population or from Holstein populations of other regions, such as Australia, New Zealand or the United States.

The classical inheritance model in quantitative genetics divides the breeding value into parent average and a Mendelian sampling term. The advantage of GS is that the latter can be inferred without its own or progeny performance [30]. In general, LD information contributes to both parts of the breeding value, and thus the accuracy due to LD is not necessarily the accuracy to predict Mendelian sampling terms. This accuracy is of great interest in order to evaluate future inbreeding and effective selection intensity when selecting on GEBVs. For this purpose and to test to what extent the accuracy due to LD obtained in this study corresponds to the accuracy to predict Mendelian sampling, cross-validations should be conducted with Mendelian sampling terms estimated from DYDs of bulls and yield deviations of dams.

The persistence of the accuracy due to LD over generations might depend on the source of LD that is utilized in estimating SNP effects, which should also be analyzed in further studies. Muir [14] showed that accuracy of GEBVs is not only persistent due to historic mutations and drift, but also when LD originates only from recent drift and selection. Furthermore, when selecting on GEBVs both the extent of LD between SNPs and QTL and the size of the QTL effects determine the fixation of QTL alleles [31] and thereby a possible decay of accuracy due to LD over generations.

### Inference of the genetic model

The number of QTL affecting a quantitative trait was estimated by Hayes et al. [32] to be in the range of 100-200. Goddard [33], however, pointed out that there are probably many more, because there is a limit to the size of the effect that can be detected. These findings are consistent with conclusions from GS studies [10,12], namely, that there are only a few major genes, but many with a small effect. Results of this study confirm these conclusions because BayesB did not perform much better than G-BLUP in the accuracy of GEBVs. BayesB was even inferior to G-BLUP for somatic cell score with a training size of 1,048 bulls. In simulations [1,2], however, in which the genetic variance was mainly determined by a few QTL with a large effect, BayesB utilized LD information considerably better than G-BLUP. The question arises why G-BLUP was mostly as good as BayesB and superior to P-BLUP despite the underlying prior assumptions for SNP effects, causing strong shrinkage. Goddard [33] pointed out that GS works in part by using deviations of the realized relationships from that expected from the pedigree, where these deviations are only useful if there is LD between SNPs and QTL or cosegregation. Those deviations seem to be estimated better if more SNPs are fitted in the model and therefore G-BLUP has advantages compared to BayesB if SNP effects and/or LD are small. This may explain why G-BLUP worked better than BayesB for somatic cell score with 1,048 training bulls. However, if more SNPs are fitted in BayesB, e.g. by altering *π *to 5 or 10%, that difference may disappear. The accuracy due to LD gives more insight into the differences of the genetic determination of quantitative traits. Because this accuracy was higher for fat and milk yield than for protein yield and somatic cell score, milk and fat yield are determined either by QTL with larger effects or the LD between SNPs and QTL is higher than for protein yield and somatic cell score. However, heritability of somatic cell score is lower than that of the yield traits [19], reducing the ability to detect QTL. One reason for the difference between protein and the other two yield traits may be *DGAT1 *[34,35], but this locus is already well estimated with the lower training size and thus the increasing difference with more training individuals results most likely from the fact that more QTL are detected.

### Comparison with simulation results

Meuwissen et al. [1] fitted 2-SNP haplotypes with BayesB and obtained an accuracy for the offspring of training individuals of 0.85 and 0.75 based on 2,200 and 1,000 training individuals, respectively. Solberg et al. [13] and Habier et al. [2,27], in contrast, fitted single SNPs and found an accuracy of 0.7 with 1,000 training individuals, where the accuracy due to LD was estimated to be 0.55 [2]. Although training data sets were comparable in size to this study, accuracies from simulations tended to be higher, which might have two main reasons. First, in simulations every offspring had two parents in the training data set so that the additive-genetic relationship information between training and validation data sets is expected to be higher at first sight, but more half sib relationships are present in real cattle populations. Second, there might be a discrepancy between the simulated genetic models and the genetic architecture (number of QTL, distribution of QTL effects, LD structure) in real populations, which might explain the lower accuracy due to LD estimated in this study. To analyze the causes of the different results between simulations and real experiments in more detail, simulations should be conducted using the real pedigree, as done by [26,36].

The decay of accuracy with *a*_*max*_, especially for BayesB, was similar to that observed in simulations over generations without further phenotyping after training [1,2,14]. In simulations, the additive-genetic relationship with training individuals is halved each generation and therefore *a*_*max *_values of the first four simulated generations after training correspond to those specified in this study. Thus, the decay of accuracy with *a*_*max *_might point to the decay of accuracy in generations after training when phenotyping is stopped. Note, however, that the number of relatives in training at a certain *a*_*max *_is different from simulations.

The differences between BayesB and G-BLUP in accuracy due to LD confirm simulation results [2], but they tended to be higher in this study than in [2]. The reason may be that 40,588 SNPs were utilized here to calculate the genomic relationship matrix used in G-BLUP, whereas only 1,000 SNPs were used in [2]. This indicates that too many SNPs dilute LD information as shown by Fernando et al. [37]. Thus, as SNP density increases in the future, the genomic relationship matrix may be less valuable than using the current density unless the training data size increases largely (see also Goddard [33]) and/or SNPs are pre-selected based on other methods such as QTL fine mapping approaches that exploit both LD and cosegregation [38].

### Comparison with other GS studies

GEBVs were combined in other GS studies analyzing real data with pedigree-based EBVs by using selection index theory [12], which increases the proportion of additive-genetic relationship information in GEBVs. In this study only direct GEBVs were considered to determine the impact of additive-genetic relationships captured by SNPs.

Accuracies of combined GEBVs in those studies should be higher, but conversely the decay of accuracy with *a*_*max *_is also expected to be larger. Further difficulties for meaningful comparisons are different numbers of training bulls and that no information about the additive-genetic relationships between training and validation bulls was provided by the other authors. However, VanRaden et al. [10] also presented squared correlations between GEBVs and DYDs using 3,500 training bulls. For the traits milk yield, fat yield, protein yield and somatic cell score correlations obtained by G-BLUP were 0.68, 0.65, 0.68 and 0.61, respectively. Correlations found for *a*_*max *_= 0.6 and 0.49 (Figure 3) were somewhat lower, which may be due to a smaller training size of 2,096 bulls. However, Hayes et al. [12] reported an accuracy of 0.67 for protein yield using G-BLUP and only 798 training bulls. Because the accuracy for protein yield with G-BLUP and 1,048 training bulls was 0.6 in this study, the relatively high accuracy estimated in [12] might indicate the contribution of additive-genetic relationships either captured by SNPs or from pedigree-based EBVs. In contrast to this study, VanRaden et al. [10] found a lower correlation for somatic cell score with G-BLUP than with a non-linear method similar to BayesB.

The fact that SNPs capture additive-genetic relationships has to be taken into account when genomic breeding values are combined with pedigree-based EBVs in practice. Otherwise the advantages of GS with respect to inbreeding and effective selection intensity may be lower.

### Future performance testing, training intervals and methods

The acceptance of GS by breeders depends to a large extent on the level of accuracy of GEBVs. Until now, breeders mainly use progeny tested bulls with a high accuracy above 0.9, which is not yet achieved with GEBVs without information from relatives. The most realistic scenario at this moment is to use GEBVs for pre-selection of young calves in combination with a subsequent progeny testing. The latter will continuously provide relatives for training and thereby ensure the highest accuracy of GEBVs. This also means that SNP effects should be re-estimated in short time intervals to always include the latest phenotypic data. The combination of GEBVs with pedigree-based EBVs might not be the only criterion for selection as deviations from expected relationships provide additional and specific information. However, the accuracy of future cohorts can be lower than for the current ones because if bulls are selected on GEBVs and mated to the breeding population as soon as they are sexually mature, the progeny test results will not be available before the next generation is ready to be selected on GEBVs (Kay-Uwe Götz, personal communication). Consider the following situation of a possible breeding program: Suppose sons of a progeny tested bull are just born. After 1.5 years, these sons can be selected on GEBVs and then mated to the population to produce both the next breeding generation and test progeny. The accuracy of their GEBVs is expected to be as high as for *a*_*max *_= 0.6 or 0.49. Another 2.5 years later, the grand-sons become selection candidates, but the accuracy of their GEBVs should be at least as low as for *a*_*max *_= 0.249, because progeny testing lasts four years in cattle and therefore no half and full sib information will be available for these grand-sons. Consequently, the GEBV accuracy of future candidates may be lower than reported in previous studies. Most likely breeders would not accept these low accuracies, meaning that the generation interval cannot be decreased at this stage of the GS developments. However, as statistical methods improve and both SNP density and training size increase, the currently expected accuracy of future candidates may also be higher.

The better approach to predict GEBVs in the future is probably BayesB rather than G-BLUP, because as SNP density and data size increase, BayesB may be able to address more QTL such that the accuracy due to LD is higher and additive-genetic relationship information becomes less important. This was demonstrated here at least for training data size (Table 4). Furthermore, there were many adjacent SNPs with *r*^2 ^close to zero and thus QTL in between might not be picked up by SNPs. VanRaden et al. [10] compared several SNP densities by removing SNPs from the 54K panel and found an increase in accuracy with density. Another option to increase both the level of accuracy and the persistence with decreasing additive-genetic relationships might be to model cosegregation in addition to LD as proposed by [39-41]. For an average *r*^2 ^of 0.225 between adjacent SNPs, which is identical to this study, Calus et al. [42] found no clear differences in the accuracy of GEBVs between a model similar to BayesB and the approach of [39] using simulated data. The question remains, however, whether this simulation result holds in practice, because the real genetic model seems to be different from the simulated one. This can be suspected from the high accuracy of 0.8 for the offspring of 1,000 training individuals in the simulations by [42] compared to the accuracies of this study. Therefore additional information from cosegregation should be useful in practice.

## Conclusions

Additive-genetic relationships between the training individuals and a selection candidate captured by SNPs affect the GEBV accuracy of that candidate. Thus, accuracy of current candidates can vary in practice. These additive-genetic relationships must be known to provide the accuracy along with GEBVs, and SNP effects should be re-estimated in short time intervals to include the most recent phenotypic data from relatives. The accuracy of future selection candidates can be smaller than reported in previous studies because information from relatives might not be available when selection on GEBVs is possible and they can be used for breeding. The decay of accuracy with decreasing additive-genetic relationships is higher with a smaller number of training individuals. Differences in accuracy of GEBVs between G-BLUP and BayesB are small, but BayesB is much more able to exploit LD information than G-BLUP. Therefore, as SNP density and training data size increase a Bayesian model averaging approach is more suitable for GS than G-BLUP. Further studies are needed to analyze the source of LD, its possible persistence with selection and the accuracy to predict Mendelian sampling terms. Cross-validations that do not take into account the structure of the data, and additive-genetic relationships in particular, are not meaningful enough for problems in plant and animal breeding.

## Competing interests

The authors declare that they have no competing interests.

## Authors' contributions

DH raised the initial questions, coded the statistical methods, conducted the analyses and wrote the manuscript; JT conducted DNA extraction and organized SNP genotyping, FS calculated daughter yield deviations, and PL helped genotyping SNPs. GT was project coordinator, added valuable suggestions and discussed the manuscript with DH. All authors read and approved the manuscript.

## Supplementary Material

Additional file 1 **Accuracy of GEBVs in case of linkage equilibrium and unrelated training individuals**. This is a pdf file used to demonstrate that the accuracy of GEBVs approaches the accuracy of pedigree-based BLUP in case of linkage equilibrium. Click here for file

## References

[B1] Meuwissen THE Hayes BJ Goddard ME Prediction of total genetic value using genome-wide dense marker maps Genetics 2001 157 4 1819 1829 11290733 10.1093/genetics/157.4.1819 PMC1461589

[B2] Habier D Fernando RL Dekkers JCM The Impact of Genetic Relationship Information on Genome-Assisted Breeding Values Genetics 2007 177 4 2389 2397 18073436 10.1534/genetics.107.081190 PMC2219482

[B3] Gianola D de los Campos G Hill WG Manfredi E Fernando R Additive Genetic Variability and the Bayesian Alphabet Genetics 2009 183 347 363 10.1534/genetics.109.103952 19620397 PMC2746159

[B4] Malécot G Les Mathématiques de l'Hérédité Paris: Masson et Cie. vi + 1948 63

[B5] de Roos APW Hayes BJ Spelman RJ Goddard ME Linkage Disequilibrium and Persistence of Phase in Holstein-Friesian, Jersey and Angus Cattle Genetics 2008 179 3 1503 1512 10.1534/genetics.107.084301 18622038 PMC2475750

[B6] Sargolzaei M Schenkel FS Jansen GB Schaeffer LR Extent of Linkage Disequilibrium in Holstein Cattle in North America J Dairy Sci 2008 91 5 2106 2117 10.3168/jds.2007-0553 18420642

[B7] Farnir F Coppieters W Arranz JJ Berzi P Cambisano N Grisart B Karim L Marcq F Moreau L Mni M Nezer C Simon P Vanmanshoven P Wagenaar D Georges M Extensive Genome-wide Linkage Disequilibrium in Cattle Genome Res 2000 10 2 220 227 10.1101/gr.10.2.220 10673279

[B8] Schaeffer LR Strategy for applying genome-wide selection in dairy cattle J Anim Breed Genet 2006 123 218 223 10.1111/j.1439-0388.2006.00595.x 16882088

[B9] König S Simianer H Willam A Economic evaluation of genomic breeding programs J Dairy Sci 2009 92 382 391 10.3168/jds.2008-1310 19109296

[B10] VanRaden PM Van Tassell CP Wiggans GR Sonstegard TS Schnabel RD Taylor JF Schenkel FS Invited Review: Reliability of genomic predictions for North American Holstein bulls J Dairy Sci 2009 92 16 24 10.3168/jds.2008-1514 19109259

[B11] Harris BL Johnson DL Spelman RJ Genomic selection in New Zealand and the implications for national genetic evaluation Proc Interbull Meeting, Niagara Falls, Canada 2008

[B12] Hayes BJ Bowman PJ Chamberlain AJ Goddard ME Invited review: Genomic selection in dairy cattle: Progress and challenges J Dairy Sci 2009 92 2 433 443 10.3168/jds.2008-1646 19164653

[B13] Solberg TR Sonesson A Wooliams J Meuwissen THE Genomic Selection using different marker types and density Proc 8th World Congr Genet Appl Livest Prod BeloHorizonte, Brazil 2006

[B14] Muir WM Comparison of genomic and traditional BLUP-estimated breeding value accuracy and selection response under alternative trait and genomic parameters J Anim Breed Genet 2007 124 342 355 18076471 10.1111/j.1439-0388.2007.00700.x

[B15] Matukumalli LK Lawley CT Schnabel RD Taylor JF Allan MF Heaton MP O'Connell J Moore SS Smith TPL Sonstegard TS Van Tassell CP Development and Characterization of a High Density SNP Genotyping Assay for Cattle PLoS ONE 2009 4 4 e5350 10.1371/journal.pone.0005350 19390634 PMC2669730

[B16] Scheet P Stephens M A fast and flexible statistical model for large-scale population genotype data: Applications to inferring missing genotypes and haplotypic phase Am J Hum Genet 2006 78 629 644 10.1086/502802 16532393 PMC1424677

[B17] Purcell S Sham P Daly MJ Parental phenotypes in family-based association analysis Am J Hum Genet 2005 76 249 259 10.1086/427886 15614722 PMC1196370

[B18] VanRaden PM Wiggans GR Derivation, Calculation, and Use of National Animal Model Information J Dairy Sci 1991 74 8 2737 2746 1918547 10.3168/jds.S0022-0302(91)78453-1

[B19] Liu Z Reinhardt F Bunger A Reents R Derivation and Calculation of Approximate Reliabilities and Daughter Yield-Deviations of a Random Regression Test-Day Model for Genetic Evaluation of Dairy Cattle J Dairy Sci 2004 87 6 1896 1907 15453507 10.3168/jds.S0022-0302(04)73348-2

[B20] Gilmour AR Gogel BJ Cullis BR Welham SJ Thompson R ASREML User Guide Release 1.0 VSN International Ltd, Hemel Hempstead, UK 2002

[B21] Fernando RL Genetic evaluation and selection using genotypic, phenotypic and pedigree information Proc 6th Wld Cong Genet Appl Livest Prod 1998 26 329 336

[B22] Vanraden PM Tooker ME Methods to explain genomic estimates of breeding value J Dairy Sci 2007 90 Suppl 1 374 (Abstr.).

[B23] Garrick DJ Equivalent mixed model equations for genomic selection J Dairy Sci 2007 90 Suppl 1 376 (Abstr.).17183106

[B24] Henderson CR Sire evaluation and genetic trends Animal Breeding and Genetics Symposium in Honor of Dr. Jay L. Lush. Champaign, IL., American Society of Animal Science and American Dairy Science Association 1973 10 41

[B25] Henderson CR Best linear unbiased estimation and prediction under a selection model Biometrics 1975 31 2 423 447 10.2307/2529430 1174616

[B26] VanRaden PM Efficient Methods to Compute Genomic Predictions J Dairy Sci 2008 91 11 4414 4423 10.3168/jds.2007-0980 18946147

[B27] Habier D Fernando RL Dekkers JCM Genomic Selection Using Low-Density Marker Panels Genetics 2009 182 343 353 10.1534/genetics.108.100289 19299339 PMC2674831

[B28] Liu Z Reinhardt F R R Estimating Parameters of a Random Regression Test Day Model for First Three Lactation Milk Production Traits Using the Covariance Function Approach Interbull Bulletin 2000 25 74 80

[B29] Liu Z Reinhardt F R R Parameter Estimates of a Random Regression Test Day Model for First Three Lactation Somatic Cell Scores Interbull Bulletin 2000 26 61 66

[B30] Daetwyler HD Villanueva B Bijma P Woolliams JA Inbreeding in genome-wide selection J Anim Breed Genet 2007 124 369 376 18076474 10.1111/j.1439-0388.2007.00693.x

[B31] Dekkers JCM Zhao H Young JM Habier D Response and inbreeding from genomic selection13th Quantitative Trait Locus and Marker Assisted Selection Workshop, Wageningen, The Netherlands, 20-21 April 2009 2009 48 http://www.qtlmas2009.wur.nl/NR/rdonlyres/F6EAFA52-C6E8-47D2-BFE5-F4BF43F7C345/84930/Book_of_abstracts_QTLMAS2009.pdf

[B32] Hayes BJ Chamberlain A Goddard ME Use of linkage markers in linkage disequilibrium with QTL in breeding programs Proc 8th World Congr Genet Appl Livest Prod BeloHorizonte, Brazil 2006

[B33] Goddard ME Genomic selection: prediction of accuracy and maximisation of long term response Genetica 2008 136 2 245 257 10.1007/s10709-008-9308-0 18704696

[B34] Grisart B Coppieters W Farnir F Karim L Ford C Berzi P Cambisano N Mni M Reid S Simon P Spelman R Georges M Snell R Positional candidate cloning of a QTL in dairy cattle: identification of a missense mutation in the bovine DGAT1 gene with major effect on milk yield and composition Genome Res 2002 12 222 231 10.1101/gr.224202 11827942

[B35] Winter A Kramer W Werner WAO Kollers S Kata S Durstewitz G Buitkamp J Womack JE Thaller G Fries R Association of a lysine-232/alanine polymorphism in a bovine gene encoding acyl-CoA:diacylglycerol acyltransferase (DGAT1) with variation at a quantitative trait locus for milk fat content Proc Natl Acad Sci USA 2002 99 9300 9305 10.1073/pnas.142293799 12077321 PMC123135

[B36] Zhong S Dekkers JCM Fernando RL Jannink JL Factors Affecting Accuracy From Genomic Selection in Populations Derived From Multiple Inbred Lines: A Barley Case Study Genetics 2009 182 355 364 10.1534/genetics.108.098277 19299342 PMC2674832

[B37] Fernando RL Habier D Stricker C Dekkers JCM Totir LR Genomic selection Acta Agric Scand A Anim Sci 2008 57 4 192 195

[B38] Croiseau P Guillaume F Fritz S Ducroq V Use of the Elastic-Net algorithm for genomic selection in dairy cattle Book of Abstracts of the 60th Annual Meeting of the European Association for Animal Production, Barcelona, Spain, August 24th-27th 2009

[B39] Meuwissen THE Goddard ME Prediction of identity by descent probabilities from marker-haplotypes Genet Sel Evol 2001 33 1009 605 634 10.1186/1297-9686-33-6-605 11742632 PMC2705394

[B40] Pérez-Enciso M Fine Mapping of Complex Trait Genes Combining Pedigree and Linkage Disequilibrium Information: A Bayesian Unified Framework Genetics 2003 163 4 1497 1510 12702692 10.1093/genetics/163.4.1497 PMC1462504

[B41] Habier D Totir LR Fernando RL A mixture genetic model for whole genome analyses Book of Abstracts of the 59th Annual Meeting of the European Association for Animal Production, Vilnius, Lithuania, 24-27 August 2008 2008 14 20

[B42] Calus MPL Meuwissen THE de Roos APW Veerkamp RF Accuracy of Genomic Selection Using Different Methods to Define Haplotypes Genetics 2008 178 553 561 10.1534/genetics.107.080838 18202394 PMC2206101

