# Supplementary material for: The impact of genetic relationship information on genomic breeding values in German Holstein cattle
Source: Genet Sel Evol. 2010 Feb 19;42(1):5. doi: 10.1186/1297-9686-42-5 (PMC2838754; doi:10.1186/1297-9686-42-5)
Supplement: Additional file 1 — Accuracy of GEBVs in case of linkage equilibrium and unrelated training individuals. This is a pdf file used to demonstrate that the accuracy of GEBVs approaches the accuracy of pedigree-based BLUP in case of linkage equilibrium. [file 1297-9686-42-5-S1.PDF]

Accuracy of genomic breeding values in case of linkage equilibrium and unrelated training individuals

The accuracy of genomic estimated breeding values (GEBVs) approaches the accuracy of pedigree-based BLUP (P-BLUP) EBVs with increasing number of SNPs fitted in the model even if SNPs and QTL are in linkage equilibrium [1]. For this to be true, it is not required that training individuals are related, but only that training and validation individuals are related. This is demonstrated below after basic definitions for linkage disequilibrium, cosegregation and additive-genetic relationships are given, and is finally completed by a simulation.

Definitions

Linkage disequilibrium (LD) is a population parameter defined as dependency between allele states at two loci on the same gamete in a natural breeding population [2]. It measures the deviation from independent sampling of allele states at different loci and can be calculated statistically as covariance between those allele states. This covariance is denoted here as D_{AB} and is calculated in general as $D_{AB} = p_{AB} - p_A p_B$, which is the difference between the frequency of gametes carrying the pair of alleles A and B at two loci (p_{AB}) and the product of the frequencies of those alleles (p_A and p_B) [3]. Note that the underlying random process is the sampling of gametes with at least two loci out of all gametes from a defined breeding population. In linkage equilibrium (LE), allele states are sampled independently, i.e., $p_{AB} = p_A p_B$, hence $D_{AB} = 0$. LD in a finite sample of individuals can be estimated as $\hat{D}_{AB} = \hat{p}_{AB} - \hat{p}_A \hat{p}_B$. In the following, D_{AB} and $\hat{D}_{AB}$ are referred to as parametric and sample LD, respectively.

Cosegregation, also called linkage, is defined as dependency between allele origin states at two loci on the same gamete. It measures the deviation from the Mendelian law of independent segregation of allele states at different loci, which occurs if loci are close enough on the same chromosome [2]. Some refer to cosegregation also as *LD within family* because allele states at two loci can be dependent if gametes are sampled within families.

Additive-genetic relationships are defined as twice the coefficient of coancestry between two individuals [4]. The latter is the probability that two gametes taken at random, one from each individual, carry alleles that are *identical by descent* [5]. The concept of *identical by descent* and thus that of coancestry is only meaningful if a base population is specified or implied [5]. The definition of additive-genetic relationships does not require that gametes contain more than one locus, and the underlying random process is fundamentally different and independent from that of LD and cosegregation.

Genome structure and sampling mode of a hypothetical population

Markers and QTL are all located on different chromosomes so that segregation of alleles at different loci is independent. Consequently, no cosegregation information is available, neither to estimate marker effects nor to predict GEBVs. Furthermore, allele states of founders are sampled independently, hence $D_{AB} = 0$ for all pairs of both loci and alleles, i.e., from a statistical point of view, the true state of nature is LE. As a result, the expected (true) marker effect, which is a function of D_{AB}, is zero.

Rationale 1: Both parametric and sample LD are zero

Trait phenotypes are available for n founders sampled from the hypothetical population, where n is greater than the total number of loci being the sum of K markers and Q QTL. Assume that in this sample $\hat{D}_{AB} = 0$ for all pairs of alleles and loci, and thus both parametric and sample LD are zero. Instead of estimating marker effects to do whole-genome selection, the marker data are exploited with a parentage test to obtain GEBVs of the founder's offspring. This parentage test is conducted by counting the number of markers at which two individuals, one from each generation, have different homozygous genotypes given that both individuals are homozygous at those loci. If the two individuals are identical homozygous at all those loci, the individual from generation 1 is declared parent of the individual from generation 2. The GEBV of individual 2 is finally calculated as the average of the trait phenotypes of all declared parents of an individual. The probability that an individual from generation 1 is falsely declared parent is $\Pi_{k=1}^{K} \frac{p_k^4+(1-p_k)^4}{[p_k^2+(1-p_k)^2]^2}$, where p_k is the allele frequency of marker k. As K increases that probability decreases, and thus the GEBV accuracy of the offspring approaches $0.5\sqrt{2h^2}$, where h^2 is the heritability of the trait. For example, let $n = 150$, $K = 100$, $Q = 10$ and $p_k = 0.25$. Then, the probability to falsely detect a parent out of those 150 founders is $2.4 \cdot 10^{-9}$.

Rationale 2: Only parametric LD is zero

Training and validation individuals

A sample of $2n$ founders is drawn from the hypothetical population, where half of them are used to estimate marker effects (training), whereas the other half is used as validation individuals being unrelated to the training data. Assume further that the training individuals are randomly mated to produce offspring that are used as related validation individuals. GEBVs of the validation individuals are obtained by using estimated marker effects. As the number of training individuals is finite, the sample LD in the training data need not be zero. In fact, the $\hat{D}_{AB}$ values for pairs of loci are distributed around the mean $D_{AB} = 0$. Consequently, estimated marker effects are not necessarily zero, but are also distributed around zero. Note further that $\hat{D}_{AB}$ for a certain pair of loci is expected to be different in each

sample, which applies here to the training and validation data set. If, conceptually, this experiment is repeated an infinite number of times, both the average sample LD as well as the average effect of a single marker is zero. From a statistical point of view, if one is interested in estimating $D_{AB} = 0$, then $\hat{D}_{AB} \neq 0$ is considered as estimation error due to random sampling of alleles. As such, the decision to refer to $\hat{D}_{AB} \neq 0$ as parametric LD would be a Type I error. From a genetic point of view, one (unknown reviewer) can argue that this random sampling is actually drift generating spurious sample LD. The question arises how useful that sample LD is to estimate GEBVs, in particular of individuals that are unrelated to the training data. Another objection is that the founders sampled from the hypothetical population actually comprise the complete finite population so that the sample LD is the true state of LD. However, this rationale could also have been started with a large but finite population, where the number of markers and QTL is smaller than the population size and all loci are in LE. The sample LD may then be interpreted in a statistical sense as estimation error.

GEBV accuracy of unrelated validation individuals

The GEBV accuracy of the founders in validation is expected to be zero, because $D_{AB} = 0$ and their allele states are sampled independently from those of the training individuals. Consequently, the accuracy of GEBVs due to LD information is zero.

GEBV accuracy of related validation individuals

The expected GEBV accuracy of the offspring can be derived in two ways. The informal way is by recognizing that the marker effects are estimated so that they explain the trait phenotypes of the training individuals. Therefore, marker effects are also useful to predict the breeding values of their offspring. As the number of markers increases, the accuracy of GEBVs approaches $0.5\sqrt{2h^2}$. The formal way can be explained as follows: Assume, for convenience of demonstration but without loss of generality, that Random-Regression BLUP (RR-BLUP) [6] is used to estimate marker effects. The equivalent model, termed G-BLUP, is an animal model in which the numerator relationship matrix, A, is replaced by the genomic relationship matrix $\mathsf{G} = \frac{\sum_{k=1}^{K} \boldsymbol{x}_k \boldsymbol{x}_k'}{\sum_{k=1}^{K} 2p_k(1-p_k)}$ [1, 7–9], where K is the number of SNPs and $\boldsymbol{x}_k$ is a vector of founder genotypes coded as the number of one of the SNP alleles. If $K \gg n$, G is a good estimate of E(G) $=$ $\mathsf{A}+$ constant [1]. GEBVs of the offspring can be directly estimated with G-BLUP by augmenting $\boldsymbol{x}_k$ with their SNP genotypes. As G approximates A well, G-BLUP approximates P-BLUP well. Therefore, the accuracy of GEBVs obtained by RR-BLUP approaches the accuracy of P-BLUP without utilizing information from neither LD or cosegregation, but only additive-genetic relationships.

Conclusions

If the parametric LD is zero, the GEBV accuracy of unrelated individuals is zero, while the accuracy of GEBVs increases with additive-genetic relationships. Thus, the parametric LD is the indicator of choice to derive the expected accuracy of unrelated individuals. Furthermore, the accuracy of GEBVs can be partitioned into accuracy due to LD information, which is the accuracy of unrelated individuals drawn from the same population as the training individuals, and accuracy due to additive-genetic relationships.

Simulation

The following simulation was conducted to demonstrate the last rationale. The training population consisted of 2,100 founders genotyped for 5,000 SNPs. The quantitative trait was simulated with heritability 0.5 and determined by 100 QTL. The QTL effects were sampled from a gamma distribution with shape 0.4 and scale 1.66 as in Meuwissen et al. [6]. Both SNPs and QTL were biallelic with allele frequency 0.5 and were all located on different chromosomes. Figure 1 depicts a histogram of r^2 between SNPs and QTL calculated from the allele states of founders, showing that r^2 values were close to zero.

Figure 1: Histogram of r^2 between simulated SNPs and QTL in the training data set

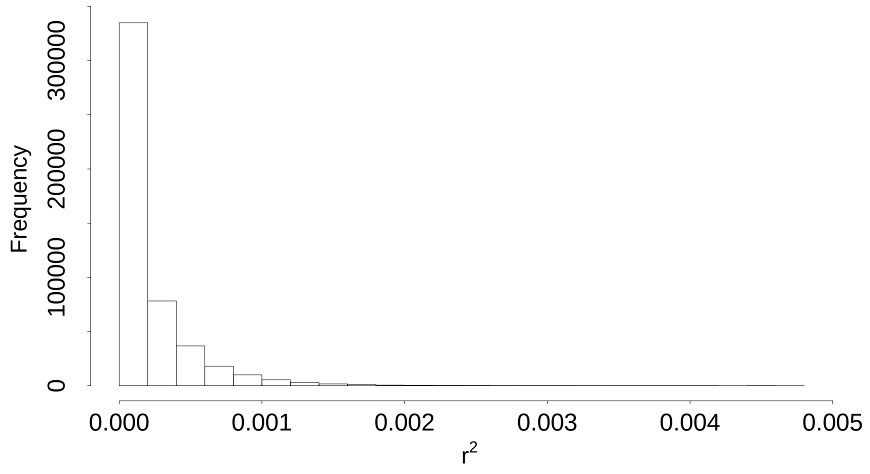

SNP effects were estimated by BayesB with $\pi = 1.0$ so that all SNPs were fitted in the model. Offspring were generated by mating each founder to exactly one other founder, and applying mendelian inheritance. GEBVs of the offspring were estimated using their SNP genotypes and estimated SNP effects. The average accuracy of these GEBVs obtained from 10 replicates of the simulation was 0.482 ($\pm$ 0.01), which is close to the expected P-BLUP

accuracy of 0.5. Furthermore, the accuracy of GEBVs for individuals that are unrelated to the training data set was close to zero. This clearly demonstrates the impact of additive-genetic relationship information.

References

1. Habier D, Fernando RL, Dekkers JCM: **The Impact of Genetic Relationship Information on Genome-Assisted Breeding Values**. *Genetics* 2007, **177**(4):2389–2397, [http://www.genetics.org/cgi/content/abstract/177/4/2389].
2. Ziegler A, Konig IR: *A Statistical Approach to Genetic Epidemiology: Concepts and Applications*. WILEY-VCH Verlag GmbH und Co. KGaA, Weinheim 2006.
3. Slatkin M: **Linkage disequilibrium - understanding the evolutionary past and mapping the medical future**. *Nat Rev Genet* 2008, **9**:477–485, [http://dx.doi.org/10.1038/nrg2361].
4. Malécot G: **Les Mathématiques de l'Hérédité**. *Paris: Masson et Cie. vi +63 pp* 1948.
5. Falconer DS, Mackay TFC: *Introduction to Quantitative Genetics*. Fourth edition. Prentice Hall 1996.
6. Meuwissen THE, Hayes BJ, Goddard ME: **Prediction of total genetic value using genome-wide dense marker maps**. *Genetics* 2001, **157**(4):1819–1829, [http://www.genetics.org/cgi/content/abstract/157/4/1819].
7. Garrick DJ: **Equivalent mixed model equations for genomic selection**. *J Dairy Sci* 2007, **90 (Suppl. 1)**:376 (Abstr.).
8. Vanraden PM, Tooker ME: **Methods to explain genomic estimates of breeding value**. *J Dairy Sci* 2007, **90 (Suppl. 1)**:374 (Abstr.).
9. Fernando RL: **Genetic evaluation and selection using genotypic, phenotypic and pedigree information**. *Proc 6th Wld Cong Genet Appl Livest Prod* 1998, **26**:329–336.
